# High frequency of pathogenic *ACAN* variants including an intragenic deletion in selected individuals with short stature

**DOI:** 10.1530/EJE-19-0771

**Published:** 2019-12-13

**Authors:** L Stavber, T Hovnik, P Kotnik, L Lovrečić, J Kovač, T Tesovnik, S Bertok, K Dovč, M Debeljak, T Battelino, M Avbelj Stefanija

**Affiliations:** 1 Unit for Special Laboratory Diagnostics, Diabetes and Metabolic Diseases, University Children’s Hospital, University Medical Centre, Ljubljana, Slovenia; 2 Department of Pediatric Endocrinology, Diabetes and Metabolic Diseases, University Children’s Hospital, University Medical Centre, Ljubljana, Slovenia; 3 Clinical Institute of Medical Genetics, University Medical Centre, Ljubljana, Slovenia; 4 Faculty of Medicine, University of Ljubljana, Ljubljana, Slovenia

## Abstract

**Context:**

Defining the underlying etiology of idiopathic short stature (ISS) improves the overall management of an individual.

**Objective:**

To assess the frequency of pathogenic *ACAN* variants in selected individuals.

**Design:**

The single-center cohort study was conducted at a tertiary university children’s hospital. From 51 unrelated patients with ISS, the 16 probands aged between 3 and 18 years (12 females) with advanced bone age and/or autosomal dominant inheritance pattern of short stature were selected for the study. Fifteen family members of *ACAN*-positive probands were included. Exome sequencing was performed in all probands, and additional copy number variation (CNV) detection was applied in selected probands with a distinct *ACAN*-associated phenotype.

**Results:**

Systematic phenotyping of the study cohort yielded 37.5% (6/16) *ACAN*-positive probands, with all novel pathogenic variants, including a 6.082 kb large intragenic deletion, detected by array comparative genomic hybridization (array CGH) and exome data analysis. All variants were co-segregated with short stature phenotype, except in one family member with the intragenic deletion who had an unexpected growth pattern within the normal range (−0.5 SDS). One patient presented with otosclerosis, a sign not previously associated with aggrecanopathy.

**Conclusions:**

*ACAN* pathogenic variants presented a common cause of familial ISS. The selection criteria used in our study were suggested for a personalized approach to genetic testing of the *ACAN* gene in clinical practice. Our results expanded the number of pathogenic *ACAN* variants, including the first intragenic deletion, and suggested CNV evaluation in patients with typical clinical features of aggrecanopathy as reasonable. Intra-familial phenotypic variability in growth patterns should be considered.

## Introduction

Short stature is a common pediatric disorder affecting 3% of the population (1) and represents one of the most frequent referrals to pediatric endocrinologists. Despite standard clinical and laboratory evaluation, the etiology of short stature is not determined in 50–90% of cases (i.e. idiopathic short stature, ISS) (2). As height is one of the most heritable human traits (3), different genetic diagnostic approaches, including next-generation sequencing (NGS), facilitated the identification of the etiology of short stature in some cases. Mutations of more than 700 genes result in growth failure, with *SHOX* haploinsufficiency being the most common underlying monogenic cause accounting for 2–3% of ISS cases (4). Recently, heterozygous mutations in the gene encoding aggrecan (*ACAN*, OMIM:*155760) has been associated with growth failure (1, 5).

**Figure 1:**
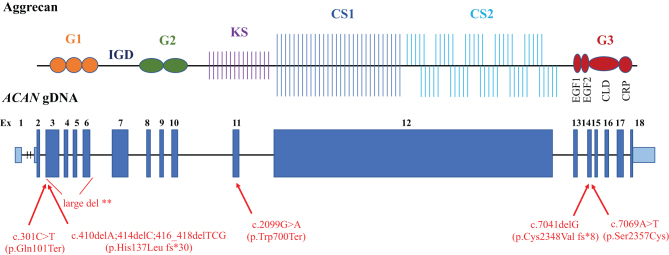
Structure of the aggrecan protein (RefSeq NP_037359.3) (6, 14) and *ACAN* gene (RefSeq NM_013227.3). Positions of the current pathogenic variants (*bottom*) with respective predicted changes in the amino acid sequence are shown. Blue boxes on the genomic DNA (gDNA) denote the coding regions (exons 1–18), drawn approximately to the related protein. Light blue boxes represent untranslated regions. G1, globular domain 1; G2, globular domain 2; G3, globular domain 3; IGD, interglobular domain; KS, keratan sulfate attachment region; CS1, chondroitin sulfate attachment region 1; CS2, chondroitin sulfate attachment region 2; EGF1, 2, epidermal growth factor-like domain 1, 2; CLD, C-type lectin domain; CRP, complement regulatory like domain; **large deletion encompassing exons 3–6 (NG_012794.1: g. 39409_45491del; NP_037359.3: p.His25_Thr350del)

Aggrecan, a large chondroitin sulfate proteoglycan, is a major structural component of the extracellular matrix of cartilage, including growth plate, articular, and intervertebral disc cartilage. Core protein comprises three globular domains (G1, G2, and G3), interglobular domain (IGD), and centrally located glycosaminoglycan attachment region (GAG) (6) (Fig. 1). G1 domain forms interactions with hyaluronan, whereas the G3 domain binds to different extracellular proteoglycans (i.e. tenascin and fibulin) via its C-type lectin repeat (CLD). GAG serves as a chondroitin and keratan sulfate attachment region, creating a highly negative-charged molecule that enables hydration of the cartilage tissue. Consequently, it allows the cartilage to withstand the high mechanical load in the skeletal joint (7).

Homozygous *ACAN* mutations lead to spondyloepimetaphyseal dysplasia, aggrecan type (SEMD, OMIM#612813) (8). Heterozygous mutations can present as spondyloepiphyseal dysplasia, Kimberley type (SEDK, OMIM#608361) (9) and short stature associated with or without accelerated bone age (BA), and an early-onset of osteoarthritis and/or osteoarthritis dissecans (OMIM#165800) (10, 11, 12).

The autosomal dominant inheritance pattern and the presence of advanced BA have been consistent features of aggrecanopathy, serving as diagnostic indicators. Recently, heterozygous *ACAN* mutations were reported in approximately 40 families worldwide (5, 10, 12, 13, 14, 15, 16), including few individuals with a decelerated BA (10, 16).

According to the recently reported clinical *ACAN*-associated features, we aimed to assess the yield of pathogenic *ACAN* variants in a study cohort having not only idiopathic short stature but also additional selection criteria. In our cohort study, the first diagnostic tier genetic analysis was NGS single nucleotide variant (SNV) analysis, whereas in patients with typical *ACAN* presentation additional copy number detection was performed.

## Participants and methods

### Participants

From 51 unrelated children and adolescents (31 females, mean age of 11 years (4–20 years)) with and without ISS (height below −2 s.d. score (SDS) (17): (i.) growth hormone (GH) deficiency, (ii.) hypothyroidism, (iii.) defined skeletal dysplasia and/or syndrome, and (iv.) cytogenetically detectable chromosomal abnormalities (e.g. Turner syndrome)), having mean height SDS −2.7 (range −2 SDS to – 4.8 SDS), referred to University Children’s Hospital of Ljubljana between 2015 and 2019, participants for the current study were selected according to the following criteria: (i.) advanced bone age and/or (ii.) autosomal dominant inheritance pattern of short stature. This cohort consisted of 16 probands (12 females, 4 males, mean age 11 years, mean height SDS −2.8) and 15 family members of *ACAN* positive probands who were additionally included in the study.

All selected patients agreed with the participation in the study. All participants or their legal guardians provided informed consent for genetic analysis. The protocol was approved by the Slovene Medical Ethics Committee (0120-36/2019/4).

### Methods

Clinical data of the probands were obtained from their electronic medical records, whereas clinical data of family members were partially available from medical history and partially from their medical documentation. Arginine and L-Dopa GH stimulation tests were performed according to previously published test procedures (18). Serum GH levels were determined by immunoassay using Immulite 2000 (Siemens). Bone age was evaluated based on Greulich and Pyle Atlas (GP) bone age determination system, 2nd edition, or determined with the BoneXpert program (19). Z-scores for height were calculated using the LMS method and the British 1990 reference growth data (17). Whole-blood EDTA samples were collected for isolation of genomic DNA according to established laboratory protocols with FlexiGene DNA isolation kit (Qiagen) (20). In all probands, next-generation sequencing (TruSight One or whole-exome sequencing (WES)) was performed. The NGS libraries for clinical exome sequencing were prepared using TruSight One sequencing panel (Illumina, San Diego, CA, USA) according to manufacturer’s instructions and sequenced on the MiSeq desktop sequencer together with MiSeq Reagent kit v3 (Illumina). WES libraries preparation and sequencing were outsourced to Novogene Co. Ltd. DNA sequencing libraries were prepared using Agilent SureSelect Human All ExonV6 kit (Agilent Technologies) following the manufacturer’s recommendations. DNA samples were fragmented to generate 180–280 bp fragments. The fragments’ overhangs were converted into blunt ends and 3′ ends adenylation enabled adapter oligonucleotides ligation in the next step. DNA fragments with ligated adapter molecules on both ends were amplified with index sequences in a PCR reaction. Exome regions were enriched with WES probes and PCR reaction amplified enriched WES target regions. After purification and WES libraries quantification, libraries were sequenced on Illumina NovaSeq 6000 (Illumina).

The collected data were analyzed with the bcbio-nextgen toolkit (https://github.com/bcbio/bcbio-nextgen) using BWA-MEM (21) to align reads to the human reference genome (GRCh37) and GATK HaplotypeCaller (22), Freebayes (23), Strelka2 (24), and VarDict (25) variant callers. A final dataset of variants was assembled from variants detected by at least two different variant callers. Standard hard filtering parameters and variant quality score recalibration according to GATK Best Practices (26, 27) recommendations were applied. FastQC was used for QC metrics and multiqc for reporting. Copy number variations in the ROI (region of interest) were inferred by CNVkit ‘Python library and command-line software toolkit’ (28). By the CNVkit algorithm, segmentation analysis and consequent targeted analysis using the moving average of the calculated copy ratio signals (smoothed trendline) within the* ACAN* gene were applied (29). Identified genetic variants with coverage >10× were annotated and filtered with VarAFT software (30).

The minor allele frequency threshold for known variants was set at 1% and all variants exceeding this value were excluded from further analysis. Candidate variants were subsequently confirmed with targeted Sanger sequencing as was family segregation analysis.

In addition, in probands in whom exome sequencing SNV analysis did not reveal causal variant in *ACAN* and who exhibited distinct *ACAN*-associated phenotype with both selected inclusion criteria, that is, accelerated BA and dominant pattern of inheritance (probands no. P1, P3, P4, and P8), additional CNV detection was performed using array CGH and NGS CNVkit detection algorithm. Array CGH analysis was performed using a commercial oligonucleotide array (Agilent 180K Baylor Oligo, Agilent Technologies) and a sex-matched human reference DNA sample (Agilent Technologies). Data were analyzed with the Cytogenomics 3.0 Software (Agilent Technologies).

To report the exact nucleotide-resolution coordinates of the detected CNV, we additionally performed long-range PCR (LR-PCR) of the selected region (exons 2–9). LR-PCR primer set was designed to the human genomic *ACAN* sequence (GRCh37/hg19) using Primer3web version 4.1.0 (31) with CGH and CNV-kit indicated deleterious sequence. LR-PCR of the selected *ACAN* region (12 729 base pairs length wild-type sequence; includes exons 2–9) was amplified using forward TTGACCTCACCATGCCTTCA and reverse TTCAGTAGGAGAGCAGGCAC primer with LongAmp Taq 2X Master Mix (thermocycling conditions: initial denaturation 30 s 94°C; 30 cycles 20 s 94°C, 20 s 60°C, 11 min 65°C; final extension: 10 min 65°C). PCR products were purified using AMPure XP Beads (Beckman Coulter Inc., Brea, CA, USA), and NGS sequencing libraries were prepared using the Nextera DNA Flex Library Prep Kit (Illumina) according to the manufacturer’s protocol and sequenced on a MiSeq sequencer (Illumina). LP-PCR NGS-sequencing data were aligned using the bwa-mem aligner, and the *ACAN* deletion was characterized using the Integrative Genomics viewer (IGV) visualization tool.

## Results

The prevalence of pathogenic *ACAN* variants in our selected study cohort was 37.5% (6/16).

Six novel *ACAN* mutations (two nonsense, two frameshifts leading to a premature stop codon, one missense, and one intragenic multi-exon deletion) in six different pedigrees (P1, P6, P10, P11, P15, and P16) (Fig. 1 and Table 1) were determined. None of the reported variants were previously reported in ClinVar, HGMD, and LOVD databases, or large population databases (GnomAD and ExAC). All variants were predicted to be damaging by different prediction algorithms (CADD, SIFT, Polyphen, and Mutation Taster). The only missense variant (c.7069A>T, p.Ser2357Cys), which is located at the G3 functional domain, inside CLD domain (Fig. 1), had the following results of *in silico* testing: CADD Phred 28, SIFT *damaging*, Mutation taster *disease-causing*, EIGEN* pathogenic*, PROVEAN *damaging*, and conservation GERP score 5.59. The missense variant was identified in proband no. 15 (P15) and segregated with short stature in the family. More precisely, it was confirmed also in her mother (P15M), who presented with short stature (−3 SDS) but was not confirmed in proband’s brother, who had a normal height (−0.62 SDS at age 5 years). Maternal grandfather was also short, but unfortunately, the segregation analysis here was not feasible, because he died before the study began.

**Table 1:** Clinical features of study probands with heterozygous *ACAN* mutation (*n* = 6) and their related *ACAN*-positive family members (*n* = 13).

Participant (no.)	Gender (f/m)	Age	Height (cm)	Height (SDS)	Bone age (SDS)	Birth weight (SDS)	Birth length (SDS)	Heterozygous mutation in *ACAN* gene**
P1	f	4y5m	97	−1.9*	+5.3	−1	−2	c.71_1051del p.His25_Thr350del
P1S	f	5y5m	109.4	−0.5	+2.5	−2.6	−2.1	c.71_1051del p.His25_Thr350del
P1F	m	46y	155	−3.4	/	+0.9	−2.5	c.71_1051del p.His25_Thr350del
P6	f	11y10m	129.7	−2.7*	−0.86	−1.3	−2.5	c.301C>T, p.Gln101Ter
P6M	f	41y	151	−2.1	/	−3	−2.6	c.301C>T, p.Gln101Ter
P6U	m	44y	150	−4.1	/	N/A	N/A	c.301C>T, p.Gln101Ter
P6GM	f	73y	140	−4.0	/	N/A	N/A	c.301C>T, p.Gln101Ter
P6BGM	m	72y	160	−2.7	/	N/A	N/A	c.301C>T, p.Gln101Ter
P10	f	13y11m	140.5	−2.9*	−0.6	−1.6	−1.5	c.7041delG, p.Cys2348ValfsTer8
P10M	f	34y	144	−3.3	/	N/A	N/A	c.7041delG, p.Cys2348ValfsTer8
P10U	m	27y	156	−3.3	/	−0.2	−1.5	c.7041delG, p.Cys2348ValfsTer8
P10GM	f	55y	151	−2.1	/	N/A	N/A	c.7041delG, p.Cys2348ValfsTer8
P11	f	14y2m	142.0	−2.8*	+0.72	−0.2	−1	c.2099G>A, p.Trp700Ter
P11S	m	10y10m	130.8	−1.8*	+1.35	−0.6	−1.5	c.2099G>A, p.Trp700Ter
P11M	f	43y	149	−2.5	/	N/A	N/A	c.2099G>A, p.Trp700Ter
P15	f	5y4m	101	−2.3	−1.59	+0.5	−0.3	c.7069A>T, p.Ser2357Cys
P15M	f	39y	146	−3.0	/	N/A	N/A	c.7069A>T, p.Ser2357Cys
P16	m	5y0m	102.6	−1.6*	+1.07	+0.2	−1	c.410_418delinsTGGA, p.His137LeufsTer31
P16F	m	36y	156	−3.3	/	N/A	N/A	c.410_418delinsTGGA, p.His137LeufsTer31

The total number of *ACAN*-positive individuals was 19.

*On GH +/− GnRH analogue therapy; **GRCh37, NM_013227.3, NP_037359.3.

BGM, brother of grandmother; F, father; f, female; GM, grandmother; m, male; m, months; M, mother; N/A not available; S, sibling; SGA, small for gestational age; U, uncle; y, years.

According to the guidelines of American Society of College of Genetics and Genomics (ACMG) (32), the nonsense, frameshift variants, and the deletion were classified as pathogenic (ACMG criteria: PVS1, PP1, PM2, and PP4), whereas the missense variant as likely pathogenic (ACMG criteria: PP1, PM1, PM2, and PP3).

**Figure 2:**
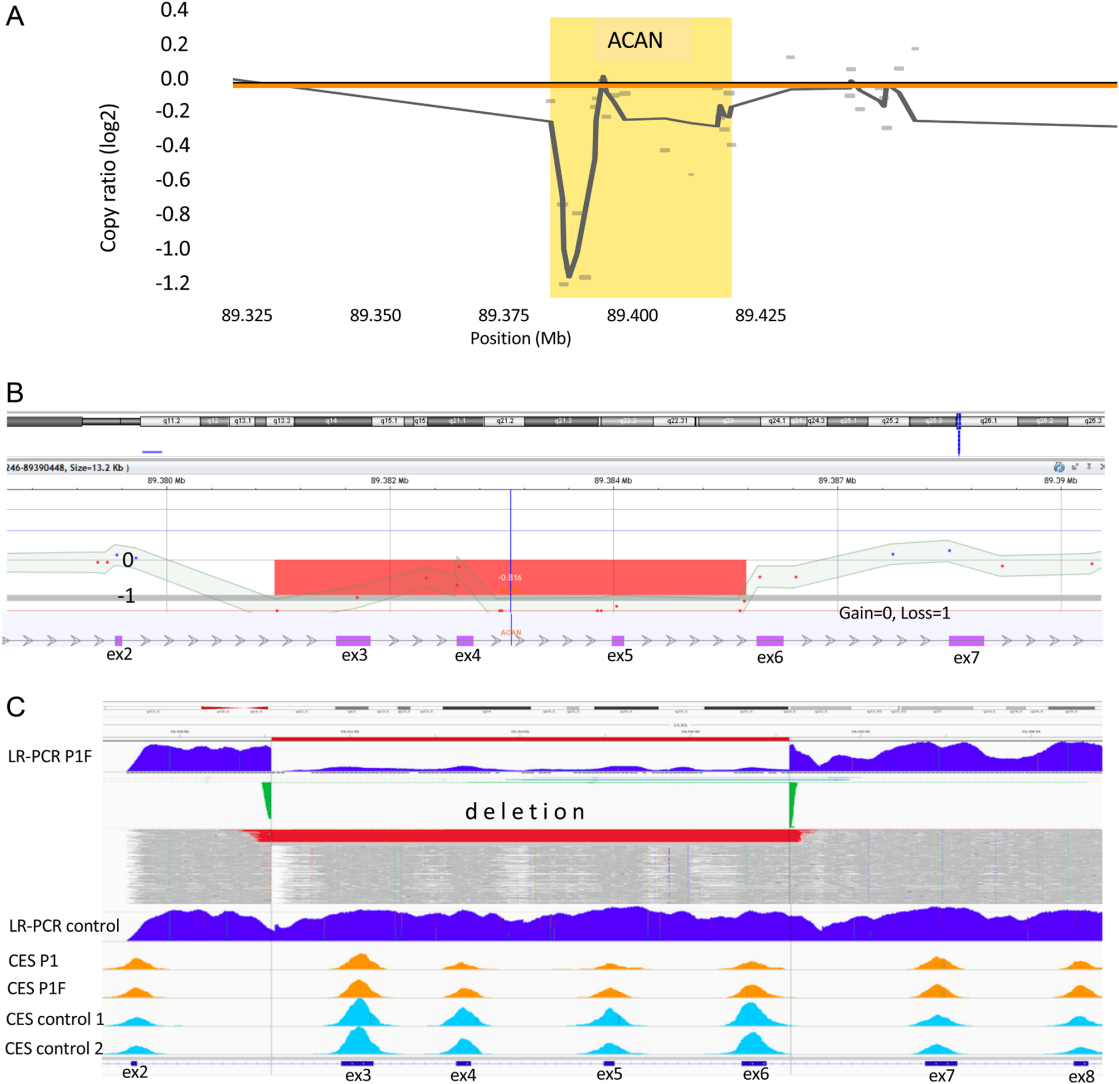
Three methods detecting heterozygous deletion in the *ACAN* gene. (A) The result of the NGS CNVkit detection algorithm indicating a possible intragenic deletion in the *ACAN* gene. (B) The result of array CGH confirming the deletion of exons 3–5 in the *ACAN* gene (arr (GRCh 37) 15q26.1 (89381207_89386488)x1). (C) LR-PCR with the NGS sequence analysis determining exact nucleotide positions of the deletion (NG_012794.1: g. 39409_45491del), encompassing exons 3–6 of the *ACAN* gene. The *red* line indicates the deletion, and *green* color marks indicate deletion coordinates. For comparison, control cases without deletion are shown. CES, clinical exome sequencing (TruSight One); P1, proband no. 1; P1F, father of proband no 1.

In probands who exhibited *ACAN*-associated phenotype (P1, P3, P4, and P8) and had negative NGS results, additional CNV detection focused on the *ACAN* gene was performed using the array CGH and NGS CNVkit algorithm (28). In P1, segmentation analysis by the CNVkit algorithm did not call a specific copy number within the *ACAN* gene, but targeted analysis using the moving average of the calculated copy ratio signals within the *ACAN* gene identified a visible drop in the copy ratio signal (Fig. 2A). The horizontal coverage of the TruSight One panel was 170×. Array CGH revealed a heterozygous 5.3 ± 1.5 kb sized deletion, encompassing exons 3–5 of *ACAN* (Fig. 2B). Additional LR-PCR of the selected region (encompassing exons 2–9 of *ACAN*) and consequent NGS analysis determined exact nucleotide positions of the deletion (NG_012794.1:g. 39409_45491del), which was 6.082 kb in size, encompassing exons 3–6 (Fig. 2C). No similar deletions or large deletions elsewhere within the *ACAN* gene were described in either genomic databases (ClinVar, Decipher, ISCA, LOVD, and HGMD) or in the large population database of healthy individuals (Database of Genomic Variants). In P3, P4, and P8, additional CNV detection analysis did not reveal any CNVs.

### Phenotypic characteristics of patients with heterozygous *ACAN* mutations

**Figure 3:**
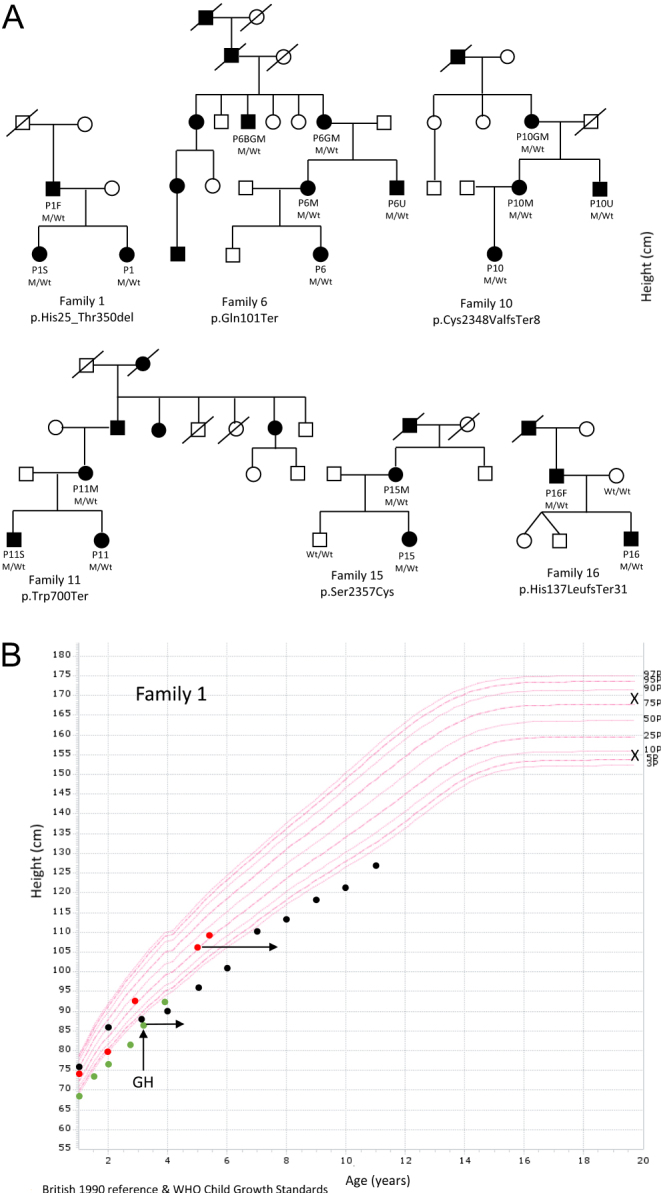
Pedigrees and growth charts. (A) Pedigrees of six unrelated families with *ACAN* pathogenic mutations. M, allele with mutation; Wt, wild type allele. (B) Growth charts of family members with a heterozygous multi-exon deletion in the *ACAN* gene. Red points indicate the growth of P1S without short stature in comparison to her father (P1F; *black points*) with early growth cessation and sister (P1; *green points*) with short stature and profound bone age advancement. The left end of each horizontal arrow represents the proband’s height at chronological age and the right end represents the bone age. Vertical arrow shows the start of growth hormone (GH) treatment. Cross signs mark mother’s (*above*) and father’s (*below*) final height. P1, proband no. 1, P1S – sister of proband no. 1, P1F, father of proband no. 1.

In total, 19 *ACAN*-positive individuals from six non-related families were identified (Fig. 3A). Their ages ranged from 3 to 73 years (Table 1). At birth, the affected individuals tended to have a lower birth weight SDS and birth length SDS (average birth weight (*n* = 11): −0.8 SDS (range: −3 to 0.9); average birth length (*n* = 11): −1.7 SDS (range: −2.6 to −0.3); Table 1). Height SDS ranged from low-normal to significant short stature (average height (*n* = 19): −2.7 SDS (range: −4.1 to −0.5); Table 1). The growth charts of *ACAN*-positive children and adolescents are presented in Supplementary Fig. 1 (see section on supplementary materials given at the end of this article). Reported variants were co-segregated with short stature phenotype in probands’ respective families, except in one family member (P1S) who had a normal height (−0.5 SDS, 30th percentile) at the age of 5 years without being on growth hormone therapy (Fig. 3B). Five probands (P1, P6, P10, P11, and P16) and one family member (P11S) were treated with GH (mean dose: 34.8 µg/kg/day, for an average of 23 months (range: 3–51 months)). The peak GH values, height gain, age at GH therapy, and duration are shown in Table 2. Only patient P16 had suboptimal GH peak levels, with normal growth factors (IGF-1: 82 µg/L (normal range: 39.0–204.5), IGFBP3 3.26 mg/L (normal range : 1.64–4.49) at age 3.8 years). P11S, who was diagnosed by segregation genetic analysis, did not perform GH stimulation tests but had normal growth factors before GH introduction (IGF-1194 µg/L (normal range: 96.9–406.6); S-IGFBP-3: 4.55 mg/L (normal range: 2.30–5.80)). In addition to GH treatment, three participants (P10, P11, and P11S) simultaneously also received treatment with GnRH analogue (triptorelin embonate 11.25 mg every 3 months). None of the treated patients achieved the final height at the time of publication. In P6, growth velocity increased significantly (height SDS increased by 1.8 SDS) during 4 years of treatment, height SDS gain was also observed during the first year follow-up in P1 (Table 2 and Supplementary Fig. 1).

**Table 2:** GH stimulation testing with arginine and/or L-dopa results and growth follow-up in all participants receiving GH.

Proband/participant	Peak GH arginine (μg/l)	Peak GH L-dopa (μg/l)	Height beforeGH (SDS)	Height with GH (SDS)	Age at GH introduction (years)	GH therapy duration (months)
P1	18.1	/	−2.5	−1.9	3.3	14
P6	7.06	12.60	−4.3	−2.7	7.9	51
P10	13.80	/	−2.5	−2.9	11.7*	27
P11	9.74	/	−2.5	−2.8	11.0*	38
P11S	/	/	−2.0	−1.8	10.6*	3
P16	5.96	6.75	−2.0	−1.6	4.5	6

*Simultaneous introduction of GnRH analogue therapy

Four out of seven children had an advanced BA, whereas in the other three BA was delayed (Table 1 and Supplementary Fig. 1).

One adult patient (P1F) presented with otosclerosis with mild nonprogressive low-frequency hearing loss of 17% since his childhood years, a sign not previously associated with aggrecanopathy.

Although in the majority of patients articular problems started in early adulthood or late adolescence, the proband no. 10 (P10) presented with frequent patellar luxations since the age of 10 years and subject no. P1F with degenerative arthritis of the spine since the age of 11 years. Not all study individuals had obvious orthopedic problems. In some families, mild facial dysmorphism was reported (mild midface hypoplasia, flat nasal bridge, and frontal bossing). Significant brachydactyly was seen in two individuals. Phenotypic characteristics of the* ACAN*-positive group are summarized in Tables 1 and 3.

**Table 3:** *ACAN*-positive individuals with skeletal findings.

Subject (No.)	Cong. elbow deformity	Cong. feet deformity (varus/valgus)	Early onset OA	Patellar (sub)luxation	Spine deformity	Knee deformity	Brachydactyly	Other
P1	−	−	−	−	+	−	−	−
P1F	−	+	+	−	+	−	−	−
P6	−	−	−	−	−	−	−	Dislocation of caput radii
P6M	−	−	−	+	−	−	−	−
P6U	+	−	−	+	−	+	+	−
P6GM	+	−	+	−	+	−	+	Multiple intervertebral disc herniations
P6BGM	+	−	−	+	−	+	−	−
P10	−	−	+	+	−	−	−	OCD
P10U	−	+	+	+	+	−	−	Radius curvature
P10GM	−	−	−	−	−	−	−	Chronic shoulder pain
P11S	−	−	−	−	−	−	−	Subluxation of prox interphalang. joint
P11M	−	−	−	−	+	−	−	−
P15M	−	−	−	−	−	−	+	−

Cong., congenital; OA, osteoarthritis; OCD, osteochondritis dissecans.

## Discussion

Recently, heterozygous *ACAN* mutations were identified as a cause of ISS with a prevalence of 1.4–6% (1, 5, 33). As the autosomal dominant inheritance pattern and the presence of advanced BA have been reported as possible diagnostic indicators of aggrecanopathy (5, 12, 14, 15, 16), our study cohort was selected according to these inclusion criteria from a larger cohort of 51 patients with ISS. The total yield of *ACAN* pathogenic variants in our study group was 37.5% (6/16). With this kind of selection, we potentially lost probands with *de novo* mutations, which are assumed to be rare. Our data suggest that pathogenic variants in *ACAN* are a common cause of familial short stature.

All our *ACAN*-positive families, except families no.1 and 15, had a nonsense/frameshift mutation with the introduction of the premature stop codon (PTC), leading to a loss of the CLD domain, which is needed to link the aggrecan molecule to other components of the extracellular matrix. Thus, these variants are likely to significantly perturb protein function. Nevertheless, to determine whether these particular mutations result in nonsense-mediated decay (NMD) of mRNA or allow translation of truncated protein as the main mechanism, additional functional studies are needed. To date, only a few *in vivo* animal models of aggrecan mutation leading to PTC were studied but each of them showed different disease mechanisms (mouse *cmd/cmd-bc*, chick *nanomelia*, dexter cattle) (34, 35, 36, 37, 38). In family no. 1, the deletion of exons 3–6 caused the lack of the whole G1 domain, which is crucial for interactions with hyaluronan and link proteins; thus, the function of the protein is likely significantly perturbed. The proposed effect for aggrecan missense mutations located in the G3 domain is the secretion of a mutant aggrecan, disrupting cartilage structure and function (dominant-negative effect) (8, 11, 39).

We herein report the first intragenic deletion in the *ACAN* gene. In one out of four patients with typical clinical presentation of aggrecanopathy (i.e. advanced bone age and familial short stature with/without early-onset articular findings) and negative exome sequencing SNV analysis result, a heterozygous deletion in *ACAN* was identified by the array CGH and detected also by the CNVkit detection algorithm using the moving average approach, but not by the default segmentation analysis. Exact coordinates of the revealed deletion were determined with additional LR-PCR and subsequent NGS analysis. To date, intragenic pathogenic *ACAN* deletions have not yet been reported. Ristolainen *et al.* report incidentally revealed a 57 bp in-frame deletion within exon 12 of *ACAN* gene in one patient with lymphoma, without functional characterization or data about patient’s growth (40), while our pedigree with intragenic deletion demonstrated several clinical features of aggrecanopathy. In patients where *ACAN* defect is clinically suspected and NGS analysis shows a negative result, additional CNV detection focused on the *ACAN* gene could be performed, with the awareness that array CGH and CNV detection pipeline for enrichment-based NGS libraries have limited sensitivity (41).

The principal clinical feature of aggrecanopathy is short stature. It is of interest to emphasize that our P1S subject with heterozygous deletion at the time of genetic evaluation did not present with short stature. Moreover, her growth velocity during early childhood increased, starting at the 0 percentile at birth and increased to the thirtieth percentile at the age of three without any therapy. Her growth pattern differed from her younger sister (P1) and father (P1F) carrying the same *ACAN* deletion (Fig. 3B), suggesting that additional genetic and environmental factors affected her growth. To date, height within the low-normal range was reported only in patients with heterozygous missense *ACAN* variants, but not in untreated patients with null variants (14). Though the growth pattern of P1S was unusual for aggrecanopathy at her prepubertal age, her BA was typically advanced, which could predict shorter final height.

Concerning other clinical features of aggrecanopathy, not all our probands presented with advanced BA. As described in previous reports, advanced BA is an indicator of the presence of *ACAN* pathogenic variants, but it is not by itself a reliable selection criterion (10, 16).

Although joint problems are presumed to commonly start in late adolescence or even later (14), our P10 presented with frequent patellar luxations already at the age of 10 years. On the contrary, the affected mother of P10 (P10M) did not show any skeletal or articular features by the time of publication (age of 34 years), indicating wide phenotype variability within the same family. Recently, it was proposed that heterozygous null variants in the upstream half of the gene had a primary effect on growth plate cartilage, whereas those in the downstream half of the gene affected both, articular and growth plate cartilage (5, 12). As the joint disease occurred also in our patients (P1F, P6, P6M, P6U, and P6GM) carrying pathogenic null variants in the upstream half of the gene, the genotype-phenotype correlation becomes more complicated, as it has been already described in some other studies (1, 14).

One adult patient (P1F) presented also with otosclerosis with consequent conductive mild hearing loss since his childhood years. The hereditary nature of otosclerosis has been acknowledged for over a century but without a precise genetic basis ascertained (42). To date, otosclerosis has not yet been reported in patients with pathogenic *ACAN* variants. Nevertheless, several reports and family linkage analyses have identified the association between 15q26.1-qter locus and otosclerosis (OTSC1) with *ACAN* to be one of the candidate genes (43, 44). Experiments on mice also showed that *Acan* is expressed in mouse auditory tissue (44). Therefore, otosclerosis may be a part of aggrecanopathy in patients with pathogenic variants. However, additional analysis of larger cohorts is required to determine the frequency of otosclerosis and hearing loss in patients with aggrecanopathy.

From four *ACAN* positive study individuals treated with GH for more than 1 year, two (P1 and P6) had a significant improvement in SDS height. The subjects P10, P11, and P11S started GH therapy during puberty after 10 years of age, which was relatively late; furthermore, they were receiving combination therapy with GnRH analogue and by the time of publication did not yet reach the final height. On the other hand, the probands P1 and P16, who started GH therapy before 5 years of age, had a short follow-up at the time of publication to assess the efficacy. In the largest clinical study of *ACAN* patients, Gkourgianni *et al.* reported that some patients tend to lose height continuously with age, whereas some can maintain their height percentile during childhood with subsequently obvious growth cessation seen in puberty, not before (14). Therefore, response to growth hormone therapy may be difficult to judge in individual *ACAN* patients because of different growth patterns during childhood/prepubertal/pubertal periods.

In conclusion, our findings corroborated the postulation that pathogenic *ACAN* variants are a common cause of familial short stature. High yield of pathogenic variants identified in our study cohort was likely related to the use of specific selection criteria, which could be suggested for a personalized approach to genetic testing of the *ACAN* gene in clinical practice. Our results expanded the number of pathogenic *ACAN* variants; furthermore, we reported the first intragenic deletion, detected by array CGH as well as by analysis of the NGS data on the exome level. Our clinical evaluation indicated that heterozygous pathogenic variants in *ACAN* most often present with evident familial short stature with or without advanced BA. However, we described a pediatric patient with an atypical growth pattern for aggrecanopathy, indicating complex genotype-phenotype correlation and quite a prominent phenotype variability among patients with identical *ACAN* genetic variants.

## Supplementary Material

Supplementary Figure 1: Growth charts of individuals with heterozygous ACAN mutations. The horizontal arrows indicate bone age estimations. Vertical arrows indicate start of GH with or without triptorelin treatment. Cross signs mark mother’s ( ) and father’s ( ) final height.

## Declaration of interest

The authors declare that there is no conflict of interest that could be perceived as prejudicing the impartiality of this study.

## Funding

The study was supported by the financial support from the Slovenian Research Agency (research core funding No. P3-0343) and University Medical Center Ljubljana Research Project (research core funding No. 20170122).

## Author contribution statement

S L, A S M, and B T contributed to the study concept and design. K J, T T, D M, and S L performed the molecular genetic analysis and data analysis with interpretation. L L did the array-CGH analysis and interpretation. S L, K P, B S, D K, and A S M selected the patients for study cohort and collected their clinical data. S L drafted the paper, and A S M along with all authors contributed to the finalizing of the manuscript. A S M is the guarantor of this work and, as such, had full access to all the data in the study and takes responsibility for the integrity of the data and the accuracy of the data analysis.
