# Supplementary figures and images for: High frequency of pathogenic ACAN variants including an intragenic deletion in selected individuals with short stature
Source: Eur J Endocrinol. 2019 Dec 13;182(3):243–53. doi: 10.1530/EJE-19-0771 (PMC7087498; doi:10.1530/EJE-19-0771)

Height (cm)

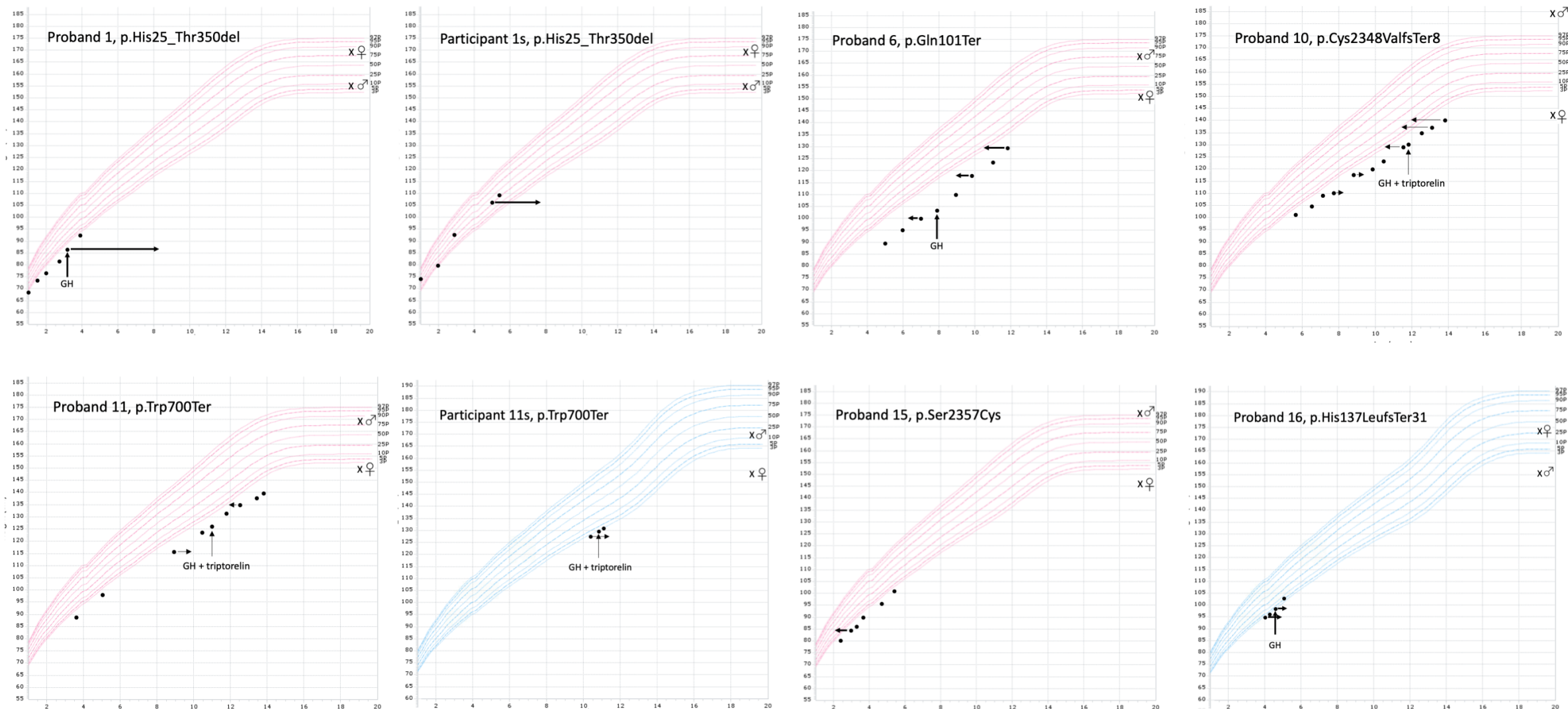

Age (years)

British 1990 reference & WHO Child Growth Standards

Supplement: Supplementary Figure 1: Growth charts of individuals with heterozygous ACAN mutations. The horizontal arrows indicate bone age estimations. Vertical arrows indicate start of GH with or without triptorelin treatment. Cross signs mark mother’s ( ) and father’s ( ) final height. [file supplementary_figure_1.pdf]
